# More competent informal caregivers reduce advanced cancer patients' unplanned healthcare use and costs

**DOI:** 10.1002/cam4.7366

**Published:** 2024-06-13

**Authors:** Louisa Camille Poco, Chetna Malhotra

**Affiliations:** ^1^ Lien Centre for Palliative Care Duke‐NUS Medical School Singapore Singapore; ^2^ Program in Health Services and Systems Research Duke‐NUS Medical School Singapore Singapore

**Keywords:** advanced cancer, cancer caregivers, caregiver competency, unplanned healthcare costs, unplanned healthcare use

## Abstract

**Background:**

Patients with metastatic cancer experience high healthcare use and costs, most of which are unplanned. We aimed to assess whether patients with more competent informal caregivers have lower unplanned healthcare use and costs.

**Methods:**

This study used data from a prospective cohort of patients with solid metastatic cancer. Patients and their informal family caregivers were surveyed every 3 months until patients' death. Patients' unplanned healthcare use/costs were examined through hospital records. Caregivers responded to the 4‐item Caregiver Competence Scale. First, in a deceased subsample of patients and their caregivers, we used patients' last 2 years of data (226 dyads) to assess the association between caregivers' competency (independent variable) and patients' unplanned healthcare use/costs (outcomes). Next, in a prospective sample of patient‐caregiver dyads (up to 15 surveys), we assessed whether patients' functional well‐being and psychological distress moderated the association between caregivers' competency and unplanned healthcare use/costs (311 dyads).

**Results:**

In the deceased subsample, during last 2 years of patients' life, caregivers' higher competency lowered the odds of patients' unplanned healthcare use [OR (CI) = 0.86 (0.75, 0.98), *p* = 0.03], and was associated with a significant reduction in unplanned healthcare costs [Coeff (CI) = −0.19 (−0.36, −0.01), *p* = 0.03]. In the prospective sample, patients' functional well‐being and psychological distress moderated the association between caregivers' competency and patients' unplanned healthcare use/costs.

**Conclusion:**

With deterioration in patients' condition and an increase in caregiving demands, improving caregivers' competency can reduce patients' unplanned healthcare use and costs. This should be further tested in future trials.

## Graphical abstract

Towards the end of life, cancer patients use unplanned healthcare which can be potentially preventable. This paper shows that as patients' condition deteriorate, caregivers play an important role in reducing unplanned healthcare use and costs, thus also potentially contributing to reducing the economic burden of cancer.

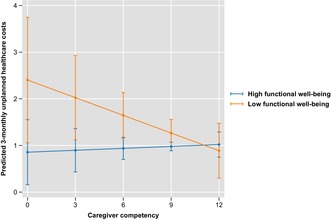

## 1 INTRODUCTION

Patients with advanced cancer incur high unplanned healthcare use and costs.^1^, ^2^, ^3^, ^4^, ^5^ Towards the end of life, three out of four cancer patients use unplanned healthcare, imposing substantial economic costs amounting to about £28 million.^4^ Much of this is potentially preventable.^5^, ^6^, ^7^ Informal family caregivers (henceforth referred to as caregivers) manage cancer patients' symptoms at home and make treatment decisions for them,^8^ thus influencing patients' unplanned healthcare use.

Caregivers' competency is an important, yet understudied, attribute that may influence their patients' unplanned healthcare use. This may happen through several mechanisms. First, more competent caregivers may effectively seek information to manage their patients' care plans at home and recognize and respond adequately to patients' symptoms and other needs, thus reducing the likelihood of patients having an adverse event and seeking unplanned healthcare.^9^, ^10^, ^11^, ^12^, ^13^, ^14^ Second, more competent caregivers may also be better able to effectively perform their roles such as communicating with healthcare providers and coordinating patients' healthcare.^15^ Third, more competent caregivers may be able to manage their caregiving activities with their other established roles and responsibilities including employment.^16^ As a result, they experience lower levels of burden and distress despite spending more hours in caregiving activities,^14^, ^17^ thus providing them with the psychological resources to manage their patients better at home.^18^ Studies further show that patients of caregivers with lower burden and distress have lower use of healthcare services,^19^, ^20^, ^21^ lower mortality/longer survival,^13^, ^14^, ^22^, ^23^, ^24^ and fewer adverse health outcomes.^25^ Overall, the empirical relationship between caregiver competency and advanced cancer patients' unplanned healthcare use, though promising and intuitive, is understudied in the scientific literature. From a policy perspective, demonstrating this relationship can inform targeted interventions for improving cancer caregivers' competency with the goal of reducing patients' healthcare costs.

We, therefore, aimed to examine the association between caregivers' competency and advanced cancer patients' unplanned healthcare use and costs. We hypothesized that patients with more competent caregivers will have lower unplanned healthcare use and costs, especially in their last years of life. We also hypothesized that patients with low functional well‐being and psychological distress will benefit more from higher caregiver competency, that is, these patients will experience a greater reduction in unplanned healthcare use and costs.

## 2 MATERIALS AND METHODS

### 2.1 Setting: Singapore

Cancer is the most common cause of death in Singapore.^7^, ^26^ Cancer patients account for 13% of total healthcare costs in the country.^27^ Towards the last 2 months of life, healthcare costs among cancer patients increase sharply driven by the increase in inpatient admissions, most of which are unplanned.^28^, ^29^ At home, caregivers are the main providers of support to patients and make treatment decisions for them, guided by cultural norms and values of filial piety.^30^ Similar to what has been found in other contexts,^31^, ^32^ advanced cancer caregivers in Singapore experience stress from the responsibilities and tasks, and report low quality of life and high burden.^33^, ^34^, ^35^, ^36^, ^37^

### 2.2 Sample

This paper used data from a cohort of patients diagnosed with stage IV solid malignancy and their caregivers from the Cost of Medical Care of Patients with Advanced Serious Illness in Singapore (COMPASS).^38^ Starting in July 2016 until March 2018, the study enrolled patients and caregivers from outpatient clinics of two major public cancer centers in Singapore. Eligible patients included those who were over age 21, were a Singapore citizen or permanent resident, were cognitively able to consent and self‐reported (determined through medical records or the Abbreviated Mental Test^39^ for participants aged above 60 years old) and had an Eastern Cooperative Oncology Group performance status ≤2.^40^

Similarly, caregivers' inclusion criteria are the following: they must be the primary caregiver of the patient, defined as one of the main persons providing care to the patient, or one of the main persons ensuring the provision of care, or the main person or one of the main persons involved in making treatment decisions for the patient. Patients' domestic helpers were excluded. A total of 311 caregiver‐patient dyads were recruited. Figure S1 in the Supplementary Materials illustrates the flow diagram of caregiver‐patient dyads included in the analysis. Patients and caregivers were surveyed every 3 months until patients' death. This study was approved by the SingHealth Centralized Institutional Review Board (2015/2781). Written consent was obtained from all patients and their caregivers.

We extracted patients' medical records containing detailed information on inpatient and emergency department visits and costs from the time of their recruitment in the study until 31 December 2021. Patient's date of death was also obtained from these records. We merged the patients' and caregivers' survey data with the billing records in the corresponding survey periods to form an analytical sample that was composed of 42 months of data.

### 2.3 Independent variable (caregiver competency)

We assessed caregivers' competence using a 4‐item measure.^18^ An example item is: “How much do you believe that you have learned to deal with very difficult situations in caring for patient?” Each item was rated on a 4‐point Likert scale (Supplementary Materials, Appendix B), and answers were reverse coded so that a higher score (range: 0–12) indicated greater competence. The scale has been shown to be reliable and valid in previous studies with caregivers of patients in palliative care.^41^

### 2.4 Outcome variables (patients' unplanned healthcare use/costs)

We used patients' healthcare use records starting 3 months after their baseline survey date. In each 3 months after the baseline and follow‐up surveys, we coded unplanned healthcare use as 1 (=any use) and 0 (=no use) based on hospital admission from or any visit to the accidents and emergency department. During the same time periods, we calculated unplanned healthcare costs as the total costs from unplanned healthcare use. Costs were adjusted for inflation to 2022 SG$ values.^42^

### 2.5 Patient undergoing active treatment

We determined if patients were undergoing any treatment (radiotherapy, chemotherapy, hormonal therapy, targeted therapy, or immunotherapy) in the 3 months prior to each survey and included this a control variable.^43^

### 2.6 Other covariates

We controlled for time‐varying patient characteristics including age; high psychological distress as measured by the Hospital Anxiety and Depression Scale (HADS) score (1 = above the 75th percentile of the score distribution; 0 = otherwise)^44^; low functional well‐being using the Functional Assessment of Cancer Therapy‐General (FACT‐G)^45^ functional well‐being subscale (1 = below the 25th percentile of score distribution; 0 = otherwise); and symptom burden using 10 items from the Functional Assessment of Chronic Illness Therapy‐Palliative Care (FACIT‐Pal) scale.^46^ We assessed patients' financial difficulties at each time point by asking them if the amount of money they had from all sources (including earnings, savings, etc.) was adequate (1) to cover the cost of treatment, (2) to take care of their daily needs, and (3) to buy little extras. Patients responded to each item as very well (=1), fairly well (=2), or poorly (=3). We calculated the financial difficulty score as the sum of responses for each item (range: 3–9), where a higher score indicated greater financial difficulty. We have used this measure previously.^47^, ^48^

We also controlled for caregiver characteristics including if a maid/helper assists with the patient's care, patient‐caregiver co‐residence, and high caregiver psychological distress (measured using HADS; 1 = above the 75th percentile of score distribution, 0 = otherwise). We assessed caregivers' financial status using the “impact on finances” subscale of the Caregiver Reaction Assessment Scale. Caregivers were asked to rate on a 5‐point Likert‐scale: (1) whether it has been difficult to pay for the patient's health expenses, and (2) if caring for the patient has put a financial strain on them. The unweighted mean score for the 2 items was summed (range: 1–5), with a higher score indicating a greater financial impact. This scale has been validated in Singapore.^49^

### 2.7 Analysis 1: Deceased patient sample and their caregivers

To represent patients with high caregiving needs, we used data from patients' last 2 years of life. We used fixed effects (FE) estimation to assess the association between caregiver competency (independent variable) and unplanned healthcare use and costs (outcomes), controlling for the time‐varying patient and caregiver variables listed above. The advantage of the fixed effects estimation is in minimizing the omitted variable bias by controlling for time‐invariant unobserved differences across the individuals. We used logistic regression models to model unplanned healthcare use. As the distribution of unplanned healthcare costs was continuous but right skewed due to the zero values, we modeled this outcome by imposing a natural logarithm transformation and estimating a linear regression model.

As a sensitivity analysis, we estimated a two‐part regression model with caregiver competency (independent variable) and unplanned healthcare costs (outcome), controlling for the time‐varying patient and caregiver variables. In the first part of this two‐part model, we fitted a logistic regression model to estimate the probability of observing a positive value versus a zero value. The second part of the model was a generalized linear model with a log link and gamma distribution specification for the positive values. The coefficients from the second part of the model were reported.

### 2.8 Analysis 2: Prospective data of patient‐caregiver dyads

Using the full prospective data allowed for variation in patients' functional well‐being and psychological distress and thus test our hypotheses on whether these factors moderated the relationship between caregiver competency and unplanned healthcare use and costs. We ran complete case FE logistic (outcome: unplanned healthcare use) and FE linear regression (outcome: unplanned healthcare costs) models including an interaction between caregiver competency (independent variable) and the two moderator variables (low functional well‐being and high psychological distress).

## 3 RESULTS

### 3.1 Patient and caregiver characteristics; full and decedent samples (Table 1)

**TABLE 1:** Summary statistics of baseline patient and caregiver characteristics.

	Full sample of dyads (*n* = 311)	Decedents (*n* = 226)
Variables	*N* (%)/Mean ± Std Dev	N (%)/Mean ± Std Dev
Patient characteristics		
Age (in years)	62.3 ± 10.2	62.4 ± 10.3
Married	254 (81.7)	184 (81.4)
Male	150 (48.2)	118 (52.2)
Ethnicity		
Chinese	238 (76.5)	174 (77.0)
Non‐Chinese	73 (23.5)	52 (23.0)
Education		
No formal education	42 (13.5)	31 (13.7)
Primary/Secondary education	177 (56.9)	131 (58)
Above secondary education	92 (29.6)	64 (28.3)
Financial difficulty score	5.9 ± 1.7	6.0 ± 1.7
If helper/maid provides care to patient	72 (23.2)	59 (26.1)
High psychological distress	59 (19.0)	68 (26.5)
Low functional well‐being	41 (13.2)	58 (25.7)
Symptom burden score	5.2 ± 5.1	5.4 ± 5.1
Cancer type		
Breast	42 (13.5)	28 (12.4)
Respiratory	91 (29.3)	63 (27.9)
Gastrointestinal	81 (26.0)	64 (28.3)
Others (Gynaecologic, Head and Neck, Skin, Genitourinary, etc.)	97 (31.2)	71 (31.4)
Undergoing active treatment	238 (76.5)	183 (81.0)
Caregiver characteristics		
Age (in years)	49.8 ± 14.5	49.8 ± 14.6
Married	245 (78.8)	176 (77.9)
Male	110 (35.4)	82 (36.3)
Ethnicity		
Non‐Chinese	77 (24.8)	54 (23.9)
Chinese	234 (75.2)	172 (76.1)
Education		
No Formal Education	6 (1.9)	6 (2.7)
Primary/Secondary education	134 (43.2)	100 (44.4)
Above secondary education	170 (54.8)	119 (52.9)
Relationship to patient		
Spouse	39 (12.5)	27 (11.9)
Parent/Parent‐in‐law	157 (50.5)	111 (49.1)
Other relative (child, sibling, etc.)	115 (37)	88 (38.9)
If caregiver lives in same house as patient	240 (77.2)	174 (77)
High psychological distress	113 (36.3)	64 (28.3)
Caregiver impact on finances score	3.0 ± 1.2	3.0 ± 1.2
Caregiver competency score	9.0 ± 2.0	8.9 ± 2.0
Outcome variables		
Unplanned healthcare use	190 (61.1)	157 (68.6)
Unplanned healthcare costs (in Singapore dollars)	1545.8 ± 8301.3	2948.7 ± 10630.6

Around half of the patients were male, while around two‐thirds of the caregivers were female. Three‐fourths of the dyads were of Chinese ethnicity. Patients (aged 34–92) were on average, 62 [SD:10] years old, while caregivers (aged 20–78) were on average 49 [SD:50] years old. Half of the caregivers cared for their parents or parents‐in‐law, and 77% co‐resided with the patient. 14% of the patients had breast cancer, 29% had respiratory cancer, 26% had gastrointestinal/colorectal/liver cancer and the rest (31%) had other cancer types (genitourinary, gynaecologic, head and neck, skin, and neurologic). Majority of the patients (77%) had received at least one of these treatments—radiotherapy, chemotherapy, hormonal therapy, targeted therapy, or immunotherapy, in the 3 months prior to the baseline survey, with chemotherapy being the most common (57%). Table S1 in the supplementary materials shows the breakdown of the treatment modalities received by patients at baseline.

Sixty‐one per cent of the patients had unplanned healthcare use at least once over the 42 months. In the sample of deceased patients, 69% had unplanned healthcare use at least once during the last 2 years of life. Throughout the 42 months, average unplanned healthcare costs for the full sample amounted to SG$ 1546 while average unplanned healthcare costs for the decedent sample amounted to SG$ 2949.

### 3.2 Analysis 1 (Table 2)

**TABLE 2:** Association of caregiver competency with unplanned healthcare use and costs (*n* = 226 dyads)^a^.

Independent Variable	Unplanned healthcare use	Unplanned healthcare costs^b^	Unplanned healthcare costs^c^
	Odds ratio	Coefficient	Coefficient
Caregiver competency	0.86**	−0.19**	−0.08**
95% CI	0.75, 0.98	−0.36, −0.01	−0.15, −0.01
*p* Value	0.03	0.03	0.03

^a^ Controlled for patient age, low patient functional well‐being, high patient psychological distress dummies, patient symptom burden, caregiver high psychological distress dummy variable, co‐residence with patient and if another helper assists with patient, patient financial difficulty, caregiver impact on finances and if patient was undergoing active treatment, ***p < 0.01, **p < 0.05, *p < 0.1, deceased patients and their caregivers' sample.

^b^ Fixed effects linear regression, log transformed outcome.

^c^ Generalized linear model with log link and gamma distribution, second part of the two‐part model. Patient and caregiver education and caregiver relationship to patient was also controlled for in this model.

The analytic sample for analysis 1 included 226 dyads of deceased patients over the last 2 years of patients' life (Figure S1). Results showed that higher caregiver competency significantly lowered the odds of having an unplanned healthcare use [OR (95% CI) = 0.86 (0.75, 0.98), *p* = 0.03], and was associated with a significant reduction in unplanned healthcare costs [Coeff (95% CI) = −0.19 (−0.36, −0.01), *p* = 0.03]. Results from the sensitivity analysis with unplanned healthcare costs as an outcome were consistent in terms of the direction and statistical significance [Coeff (95% CI) = −0.08 (−0.15, −0.01), *p* = 0.03]. We used the Hausman test to formally test whether the FE model was the correct model specification, and results from the test (Table S2) were significant, supporting the use of the FE model.

### 3.3 Analysis 2

**FIGURE 1:**
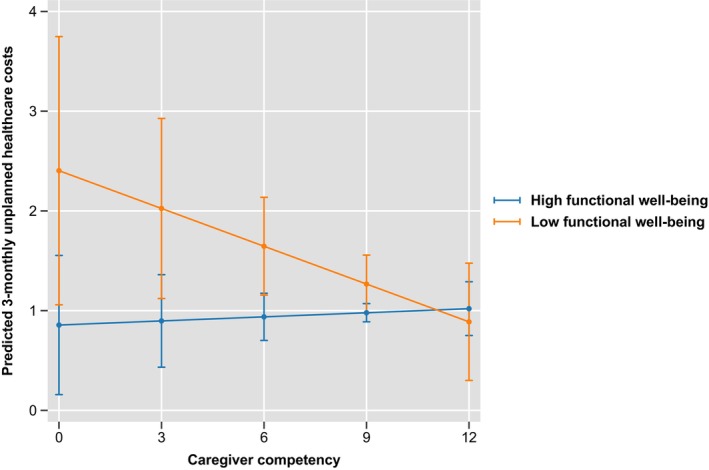
The moderating effect of low functional well‐being on the relationship between caregiver competency and unplanned healthcare costs.

**FIGURE 2:**
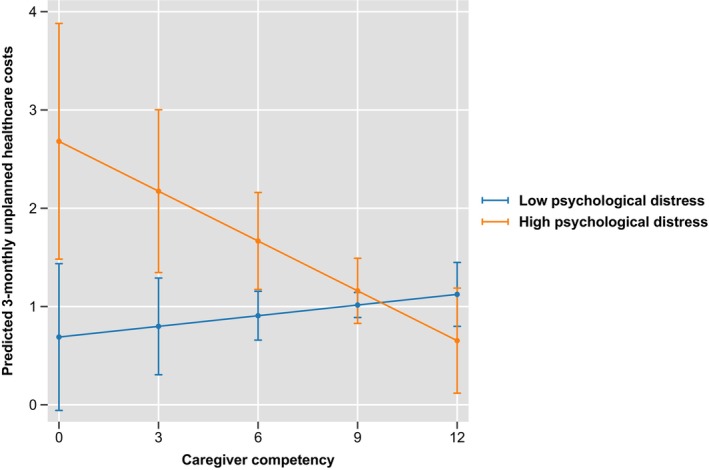
The moderating effect of high psychological distress on the relationship between caregiver competency and unplanned healthcare costs.

The analytic sample for analysis 2 consisted of full prospective data of patient‐caregiver dyads interviewed 3 monthly over 42 months (311 dyads). Results showed no significant association between caregiver competency and patients' unplanned healthcare use/costs. However, we found that patients' low functional well‐being and high psychological distress moderated the association between caregiver competency and unplanned healthcare use and costs. When patients' functional well‐being was low, higher caregiver competency was associated with lower odds of patients having unplanned healthcare use [OR (95% CI) = 0.86 (0.74,1.01), *p* = 0.07] and lower unplanned healthcare costs [Coeff (95% CI) = −0.14 (−0.30, −0.02), *p* = 0.08]. (Figure 1; Table S3) Similarly, when psychological distress was high, higher caregiver competency was associated with lower odds of patients having unplanned healthcare use [OR (95% CI) = 0.78 (0.65, 0.94), *p* = 0.01] and lower unplanned healthcare costs [Coeff (95% CI) = −0.21 (−0.35, −0.06), *p* = 0.01]. (Figure 2; Table S4). The associations were not significant with low levels of psychological distress and high levels of functional well‐being.

## 4 DISCUSSION

Our results showed that caregiver competency was associated with a lower likelihood of unplanned healthcare use and a reduction in unplanned healthcare costs. However, this association was true only when patients were very sick, that is, during the last 2 years of life, when their functional well‐being was low, or psychological distress was high. In other words, patients who experienced worsening illness trajectory were more likely to benefit from having a more competent caregiver. The findings have implications regarding caregivers' role in reducing unplanned healthcare use and costs among advanced cancer patients, especially older individuals.

A previous study using the same dataset showed that higher levels of caregiver competency moderated the relationship between time spent caregiving and caregivers' anxiety. This implied that as caregiving hours increased, caregivers with lower but not higher competency felt more anxious.^14^ Our results extend these findings by showing that as caregiving demands increase, caregiver competency also has a protective effect on patients' outcomes as measured by unplanned healthcare use. The likely pathways explaining this relationship have been discussed in the introduction.

This study has clinical implications. Advanced cancer patients' uncontrolled symptoms are one of the main drivers for their unplanned healthcare use.^5^ We show that caregivers play an important role in managing acute events at home and competent caregivers can more effectively perform this role, thus preventing patients' unplanned healthcare use.^15^ Yet, programs and interventions to manage patients' care have often not targeted caregivers. Our results support the development of interventions to equip caregivers with education and skills to manage patients at home, and psychosocial interventions such as cognitive behavioral therapy to improve self‐efficacious beliefs among caregivers. Such interventions have in the past, been shown to improve caregiver quality of life, mental health, self‐efficacy, and other caregiver outcomes.^50^, ^51^ Future trials of similar interventions should also assess patients' unplanned healthcare use as an outcome. Programs to prevent unplanned healthcare use at the end of life can also incorporate these caregiver interventions as one of their key components.

Our findings also have policy implications. Given the increasing burden of cancer and related healthcare costs and that caregivers have the potential to reduce these costs, governments can allocate funding towards the systematic provision of caregiver programs and incorporate these programs as part of patient care. Family‐friendly workplace policies can help ease the strain on employed caregivers, allowing them to care for patients at home.

Although a few papers have previously reported that caregiver factors such as burden and distress were associated with healthcare use and mortality in patients with an advanced illness,^13^, ^52^, ^53^ there has been no examination of the role of caregiver competency in reducing unplanned healthcare use and costs in cancer patients. This paper thus contributes new knowledge by establishing the relationship between caregivers' competency and patients' unplanned healthcare use using caregiver‐patient dyad data linked with medical records.

This study has limitations. First, though we employed prospective cohort data and assessed healthcare use in the 3‐month following each survey using fixed effects models to minimize any omitted variable bias, some reverse causality may still exist since patients' unplanned healthcare use and their illness may affect their caregivers' perceptions of their competency. Second, 48% of patients in our sample did not have their caregivers interviewed for the study. This may be a limitation if patients whose caregivers refused to be interviewed were systematically different from those included in our analytic sample. We do not have the characteristics of caregivers who refused to be interviewed. Finally, around 75% of patients and caregivers are of the Chinese ethnicity—this roughly represents the ethnic composition of Singapore. The external validity of our findings thus needs to be confirmed in other settings and patient‐caregiver populations.

## 5 CONCLUSION

This study shows that with deterioration in advanced cancer patients' condition and an increase in caregiving demands, improving caregiver competency has the potential to reduce unplanned healthcare use and costs. These findings highlight the role of caregivers in reducing the economic burden of cancer. Future studies should evaluate the efficacy of caregiver interventions to improve caregivers' self‐appraisal of competency, in reducing unplanned healthcare use and costs.

## AUTHOR CONTRIBUTIONS

**Louisa Camille Poco:** Conceptualization (equal); formal analysis (lead); methodology (equal); visualization (lead); writing – original draft (equal); writing – review and editing (equal). **Chetna Malhotra:** Conceptualization (equal); formal analysis (supporting); methodology (equal); supervision (lead); writing – original draft (equal); writing – review and editing (equal).

## CONFLICT OF INTEREST STATEMENT

The authors have no conflict of interest to disclose.

## Supporting information


Appendices S1–S2.

## Data Availability

The data that support the findings of this study are available from the corresponding author upon reasonable request.
