# Supplementary material for: More competent informal caregivers reduce advanced cancer patients' unplanned healthcare use and costs
Source: Cancer Med. 2024 Jun 13;13(11):e7366. doi: 10.1002/cam4.7366 (PMC11176569; doi:10.1002/cam4.7366)
Supplement: Supplementary file 1 — Appendices S1–S2. [file CAM4-13-e7366-s001.docx]

**Supplemental Materials**

**Appendix A**

**Table A1: Summary statistics of treatment modalities received by patients at baseline**

| **Variables** | **Full sample of dyads (n=311), N (%)** | **Decedents**  **(n=226), N (%)** |
| --- | --- | --- |
| *Treatment modality (dichotomous variable =1 if received this treatment modality)* | | |
| Radiotherapy | 7 (2.3) | 5 (2.2) |
| Chemotherapy | 178 (57.2) | 140 (61.9) |
| Immunotherapy | 14 (4.5) | 9 (4.0) |
| Targeted therapy | 71 (22.8) | 52 (23.0) |
| Hormonal therapy | 18 (5.8) | 14 (6.2) |
| Any therapy | 238 (76.5) | 183 (81.0) |

**Table A2: Hausman test^1^**

| **Outcome variable** | **P-values** |
| --- | --- |
| Unplanned healthcare use | 0.0002 |
| Unplanned healthcare costs | 0.0000 |

^1^Null hypothesis: preferred model is RE.

**Table A3: Association of caregiver competency on unplanned healthcare use and costs, moderator analysis 1 (n=311 dyads)^1^**

| **Independent Variables** | **Unplanned healthcare use** | **Unplanned healthcare costs^2^** |
| --- | --- | --- |
|  | Odds Ratio | Coefficient |
| Low functional well-being^3^ | 4.51* | 1.55** |
| 95% CI | 1.00,20.42 | 0.10,2.99 |
| P-value | 0.05 | 0.04 |
|  |  |  |
| Caregiver competency | 1.04 | 0.01 |
| 95% CI | 0.92,1.17 | -0.07,0.09 |
| P-value | 0.55 | 0.73 |
|  |  |  |
| Low functional well-being*caregiver competency | 0.86* | -0.14* |
| 95% CI | 0.74,1.01 | -0.30,0.02 |
| P-value | 0.07 | 0.08 |

^1^ Controlled for patient age, high patient psychological distress dummy, patient symptom burden, caregiver high psychological distress dummy, coresidence with patient and if another helper assists with patient, patient financial difficulty, caregiver impact on finances and if patient was undergoing active treatment, *** p<0.01, ** p<0.05, * p<0.1, full sample of dyads.

^2^ Fixed effects linear regression, log transformed outcome.

^3^ Below the 25^th^ percentile of the overall distribution of reported values of functional well-being subscale of the Functional Assessment of Cancer Therapy-General.

**Table A4: Association of caregiving competency on unplanned healthcare use and costs, moderator analysis 2 (n=311 dyads)^1^**

| **Independent Variables** | **Unplanned healthcare use** | **Unplanned healthcare costs^2^** |
| --- | --- | --- |
|  | Odds ratio | Coefficient |
| High psychological distress | 10.65*** | 1.99*** |
| 95% CI | 1.98,57.20 | 0.63,3.35 |
| P-value | 0.01 | 0.00 |
|  |  |  |
| Caregiver competency | 1.07 | 0.04 |
| 95% CI | 0.94,1.22 | -0.05,0.12 |
| P-value | 0.31 | 0.41 |
|  |  |  |
| High psychological distress*caregiver competency | 0.78*** | -0.21*** |
| 95% CI | 0.65,0.94 | -0.35,-0.06 |
| P-value | 0.01 | 0.01 |

^1^ Controlled for patient age, low patient functional well-being dummy variable, patient symptom burden, caregiver high psychological distress dummy variable, coresidence with patient and if another helper assists with patient, patient financial difficulty, caregiver impact on finances and if patient was undergoing active treatment, *** p<0.01, ** p<0.05, * p<0.1, full sample of dyads.

^2^ Fixed effects linear regression, log transformed outcome.

^3^ Above the 75^th^ percentile of the overall distribution of reported values of the hospital anxiety and depression scale.

**Figure A1. Flow diagram of participants**


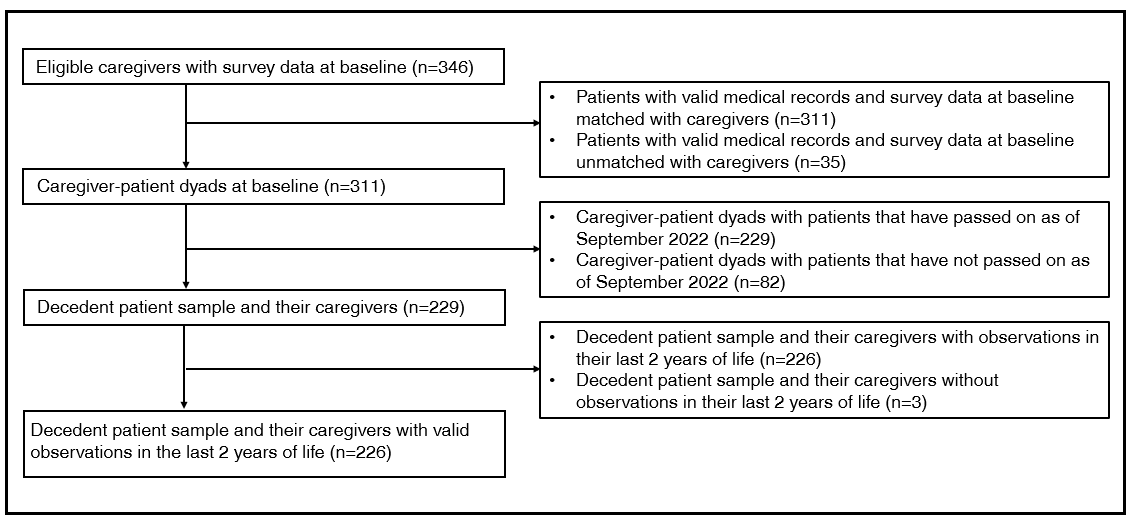


**Appendix B**

**Caregiver Competence Scale**

- How much do you believe that you have learned to deal with very difficult situations in caring for (Patient)? (e.g., seeing to physical needs of patient)

1 - Very much
2 - Somewhat
3 - Just a little
4 - Not at all

- How much do you feel that all in all, you are a good caregiver to (Patient)?

1 - Very much
2 - Somewhat
3 - Just a little
4 - Not at all

- How competent do you feel (i.e., have the skills) in providing care to (Patient)?

1 - Very competent

2 - Fairly competent

3 - Just a little competent

4 - Not at all competent

- How self-confident (i.e., sure) do you feel about providing care to (Patient)?

1 - Very confident

2 - Fairly confident

3 - Just a little confident

4 - Not at all confident
